# Comparison of the effectiveness of a serious game and pre-recorded lecture in diagnosis and treatment planning of oral lesions for dental students

**DOI:** 10.1038/s41598-024-83433-0

**Published:** 2024-12-27

**Authors:** Waranun Buajeeb, Patricia A Reynolds, Hatailux Boontub, Yanon Tangmanpuwadol, Kawin Sipiyaruk

**Affiliations:** 1 Department of Oral Medicine and Periodontology, Faculty of Dentistry, Mahidol University, Bangkok , Thailand; 2 Faculty of Dentistry, Oral & Craniofacial Sciences, King’s College London, London, England; 3 Mahidol International Dental School, Faculty of Dentistry, Mahidol University, Bangkok, Thailand; 4 Department of Orthodontics, Faculty of Dentistry, Mahidol University, Bangkok, Thailand

**Keywords:** Dental education, Interactive learning, Learning design, Serious game, Technology-enhanced learning, Diagnosis, Dental education, Dentistry, Oral medicine

## Abstract

This research evaluated the effectiveness of an online simulation-based serious game as a learning tool in diagnosis and treatment planning for oral lesions (SimOL) in comparison to a pre-recorded lecture-based approach and to determine its appropriate integration into the undergraduate dental curriculum. A crossover randomized control trial was conducted with a cohort of 77 dental undergraduates. They were randomly assigned into two groups. Group A underwent SimOL followed by a pre-recorded passive lecture, whereas Group B experienced the converse learning sequence. Pre-assessment, Post-assessment I, and Post-assessment II were administered to evaluate knowledge improvement, along with a satisfaction questionnaire to gather learner perceptions. Data were analyzed using descriptive statistics, independent t-test, and repeated measures ANOVA with the Bonferroni post-hoc test. With two dropouts, this research included 75 students (Group A: *n* = 38 and Group B: *n* = 37). The results demonstrated statistically significant score differences across the three assessments in both groups. Significant improvements in assessment scores were observed after the initial instructional approaches in both groups (*P* < 0.001). However, the additional SimOL or lecture did not significantly enhance Post-assessment II scores in either group (*P* > 0.05). No statistically significant score differences were found between Group A and Group B in all assessments (*P* > 0.05). Participants held a positive perception of SimOL. These findings demonstrated the effectiveness of SimOL in enhancing knowledge related to the diagnosis and treatment planning of oral lesions at a level equivalent to that of a pre-recorded lecture, highlighting its potential as an interactive learning tool in dental education.

## Introduction

Oral medicine provides an important role in dentistry, providing comprehensive care for patients with oral mucosal lesions. This dental discipline requires dental practitioners to have competence in diagnosing and managing oral lesions. In dental education, learning outcomes regarding oral medicine are expected for both undergraduate and postgraduate levels^1^. Various learning strategies have been implemented to support learners, including classroom settings (lectures or small group teaching) and clinical training^2,3^. The combination of these diverse learning approaches would ensure that learners could attain competence in cognitive, psychomotor, and affective domains.

Traditional learning strategies are currently facing criticism due to their inherent challenges within conventional educational settings. One of the common difficulties is the inflexibility of traditional classroom settings, in which technology-enhanced learning (TEL) could enable instructors and learners to control over location, time, and pace^4,5^. A lack of interactive components in traditional approaches, mainly one-way communication lectures, could have an adverse impact on student involvement and motivation, which can result in decreased excitement for learning, especially for new generation of learners^6–8^. Moreover, traditional teaching approaches appear to have limitations in developing critical thinking and problem-solving skills^9^. Scientific evidence also reveals that interactive TEL could enhance knowledge retention^10^. Serious games therefore should be considered to create interactive learning environments for teaching and learning in oral medicine courses.

Serious games appear to have numerous advantages, leading to an increasing use in the field of healthcare^11^, including dental education^12,13^. They allow learners to apply and validate their knowledge in meaningful learning experiences^14^. Serious games have proved as effective learning tools, where dental students could enhance their knowledge and understanding with engagement and motivation^15,16^. Furthermore, serious games could offer a safe environment for learners to improve their knowledge and skills^12^. Consequently, dental students could gain their diagnostic abilities with virtual patients^10,17^. This encourages knowledge competence before gaining experience in clinical practice and ensures that a comprehensive range of conditions can be covered.

An online simulation-based serious game has been demonstrated to have positive educational impact in teaching and learning oral medicine, where dental students could gain knowledge with engagement^10^. However, there is limited evidence that such strategies can be effectively implemented as a replacement or supplementary to a pre-recorded lecture. Therefore, this research was conducted in order to evaluate the effectiveness of online simulation-based serious game in an oral medicine course compared to a pre-recorded lecture. It also aimed to investigate how this interactive learning tool should be implemented into an undergraduate dental curriculum. This research would emphasize the significance of using serious games to bridge the gap between academic knowledge from classroom settings to its practical application in clinical practice.

## Materials and methods

### Theoretical foundation

The theoretical basis of this study is grounded in existing evidence supporting the effectiveness of serious games in educational contexts, as well as the complementary frameworks of constructivist learning theory^18^and experiential learning theory^19^. Together, these theories support the development and implementation of an online serious game in diagnosis and treatment planning of oral lesions (SimOL). The design of SimOL aligns with constructivist learning theory by immersing students in simulated situations that require them to actively construct new knowledge from their prior experiences through the interaction with virtual patients. The constructivist approach promotes deeper understanding, as students build knowledge through simulated clinical tasks rather than passive observation. Experiential learning theory further reinforces this constructivist approach by framing learning within SimOL as an iterative process of concrete experience, reflective observation, abstract conceptualization, and active experimentation. In other words, SimOL allows students to interact with virtual patients in simulated clinical scenarios, reflecting on feedback from the game system, and adjust their answers, leading to refined conceptual understanding.

### Research design

**Fig. 1:**
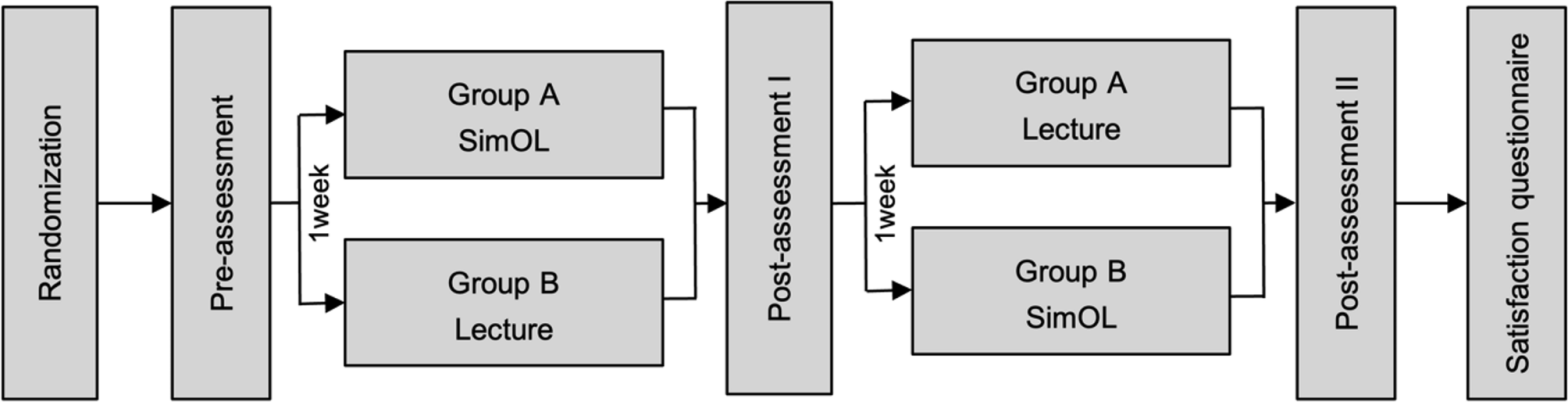
Diagram illustrating the flow of trial design.

This study employed a crossover randomized control trial to assess and compare the effectiveness of SimOL with a pre-recorded passive lecture. Both learning approaches require learners to participate for a duration of one hour. The design and report of this trial design adhered to the guideline for reporting parallel group randomized trials of the Consolidated Standards of Reporting Trials (CONSORT) guidelines^20^. All fourth-year dental students from the Doctor of Dental Surgery Program, Faculty of Dentistry, Mahidol University were subjected to SimOL as a mandatory task of an oral lesion course. They were randomly assigned to two groups with different instructional approaches. Group A was directed to employ SimOL prior to the pre-recorded lecture, whereas Group B was subjected to the converse learning design. Three paper-based tests (Pre-assessment, Post-assessment I, and Post-assessment II) were administered to all learners to evaluate their prior knowledge and how they could learn (knowledge gain) after the completion of the first and subsequent instructional methods, respectively. Two intervals of one-week washout periods were employed after each assessment to minimize a test-retest memory effect. This design investigated the implementation of SimOL in an oral lesion course, whether as a replacement for or a supplement to a traditional but pre-recorded lecture setting. In terms of ethical consideration, this method allowed learners in both groups to have similar learning experiences. Figure 1 illustrates the flow of trial design. These procedures were conducted at the Faculty of Dentistry, Mahidol University in July 2023.

### An online serious game in diagnosis and treatment planning of oral lesions

SimOL was firstly developed in 2022. The game was reviewed by three external experts in oral medicine to ensure content accuracy. SimOL was piloted in 28 fourth year students from the Dental of Dental Surgery Program (International Program), in which the findings demonstrated the learners to gain knowledge after interacting with the game^10^. Consequently, based on the positive outcomes of the previous study, the subsequent version of SimOL was incorporated as a mandatory component for a larger cohort of learners from the Dental of Dental Surgery Program in the following academic year. The SimOL activity requires an approximate duration of one hour to complete all game tasks.

SimOL is a simulation-based online serious game available on iOS platform. Within the game, leaners were allowed to choose their character, as a male or female dental students. There were three simulated patients with different oral lesions, including allergic contact stomatitis, mucous membrane pemphigoid, and pemphigus vulgaris. These lesions were purposefully chosen due to their prevalence among dental professionals, and their timely detection has potential benefits for patients. Learners needed to interact with the game through various quiz formats (single response, multiple response, and open-ended questions) in order to get information required for definitive diagnosis and treatment planning. Immediate responses and informative feedback in various formats (text information provided by a virtual instructor, facial expression of simulated patients, and a score retrieved from the game system) were designed to support users to learn from their mistakes and improve their understanding. Upon completing each learning scenario, learners can view their achievement points on a score page, reflecting their performance on various learning tasks.

**Fig. 2:**
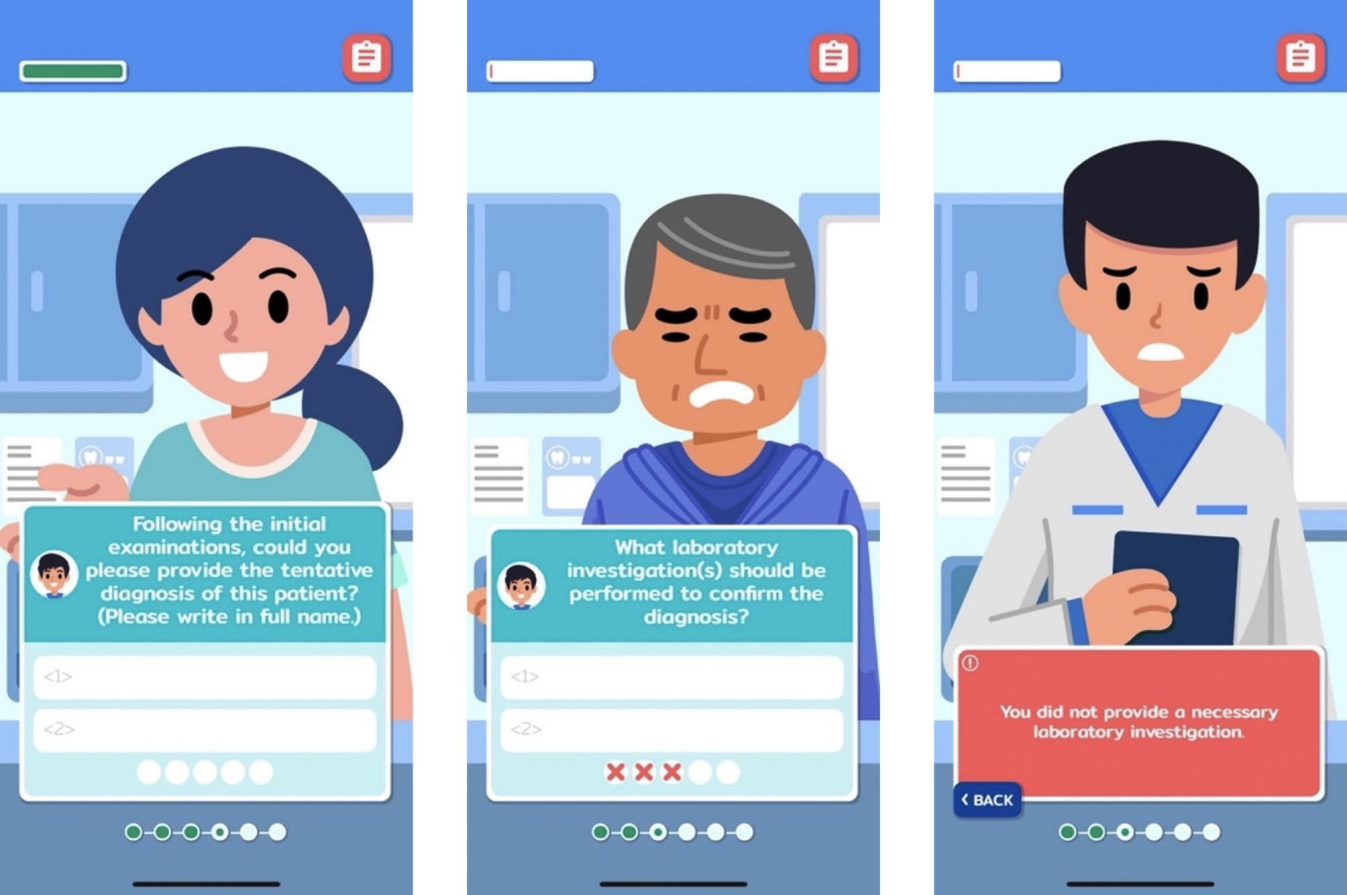
In-game screenshots of SimOL where learners could interact with the game.

In addition to the user interface, open-ended questions designed for questions regarding differential and definite diagnosis were improved following user feedback from dental students in the 2022 academic year. Although this format allowed users to freely response to the game without predetermined answer options to mimic a real situation of clinical practice, only exact spellings would be considered as correct responses which could make learners felt frustrated, based on previous findings^10^. Consequently, the answer pool was extended in this version by considering the set of answers provided by the learners of the previous cohort. Examples of in-game screenshots of SimOL are presented in Fig. 2.

### A pre-recorded passive lecture

A pre-recorded passive lecture was prepared as a conventional pedagogical approach, serving as a benchmark for comparison with SimOL. This recorded format was chosen to ensure consistent quality across both participant groups. The lecture content comprehensively addressed topics such as allergic contact stomatitis, mucous membrane pemphigoid, and pemphigus vulgaris, aligning with SimOL. The delivery of the lecture was arranged within a classroom setting, fostering synchronous participation among all learners within each respective group. Therefore, the learners restricted from fast-forwarding or replaying the content. The duration of the lecture matched the time allocated for completing the SimOL activity, ensuring equal instructional time for both methods.

### Research participants and randomization

All fourth-year dental students from the Doctor of Dental Surgery Program who enrolled in the oral lesion course during the 2023 academic year were eligible for this research. They had attended lectures on basic oral diagnosis but had not yet received specific training on oral lesions. This supported a constructivist approach and ensured cognitive readiness by preventing the game tasks from being too challenging. Students were excluded if they did not complete at least one task within the arranged schedule, as they could have been exposed to related learning content through other activities. These inclusion and exclusion criteria ensured cognitive readiness and minimized bias from varying levels of prior exposure to related content, helping to accurately reflect the impact of SimOL on student learning and satisfaction.

Based on previous randomized control trials in healthcare education^21–24^, to detect the differences between the SimOL and lecture groups with a two-sided significance level of 5% and power of 80%, a sample size of 8 to 36 participants for each group was estimated using G*power statistical software. However, as SimOL was implemented as a mandatory task of an oral lesion course, the entire cohort of 77 dental students from the 2023 academic year was incorporated into this study. Consequently, the sample size ensured the robust statistical power of the research.

The participants were randomized into two groups using a random sequence, generated by Microsoft Excel 2023. The implementation of a double-blind technique was deemed impractical for this research, given that both the students and instructors assigned to each group were cognizant of the learning approaches they received. Nevertheless, to mitigate the potential influence of bias, the research data were coded with anonymous identifiers, ensuring that the researcher responsible for assessing and analyzing the gathered data remained blinded to the allocation throughout the study. This approach additionally assured the anonymity of student data for research purposes.

### Outcome measurements

Knowledge gain from either SimOL or lecture was evaluated from score improvement retrieved from Pre-assessment, Post-assessment I, and Post-assessment II paper-based tests. Each assessment contained 15 identical multiple-choice questions to assure the level of difficulty, but questions and answer choices were arranged in random sequence to mitigate the test–retest memory effect^16,25–27^. These assessments had previously been administered to a different group of dental students in the 2022 academic year, in which the validity and reliability were performed using content validity and Kuder-Richardson Formula 20 (KR-20), respectively^10^.

The paper-based questionnaire consisted of three parts (15 five-point Likert scales), which were ‘perceived usefulness’ (5 items), ‘perceived ease of use’ (5 items), and ‘perceived enjoyment’ (5 items), with ‘1’ indicating ‘Strongly disagree’ and ‘5’ indicating ‘Strongly agree’. These aspects were designed based on the technology-acceptance model to gather user satisfaction^28^, and it was then adapted to suit the serious game evaluation^16,25,29^. Similar to the knowledge assessments, the satisfaction questionnaire had been validated in the previous academic year^10^.

### Data analysis

The research data were analyzed using the Statistical Package for Social Sciences software (SPSS, version 29, IBM Corp., Armonk, NY). Descriptive statistics were employed to offer a comprehensive overview of the dataset. A repeated measures ANOVA with the Bonferroni post-hoc test was performed to evaluate the two intervals of knowledge improvement. The differences of score improvement between the two groups were analyzed using an independent t-test. The significance level was defined at the alpha level of *P* < 0.05.

### Ethics approval

This research was approved by the Institutional Review Board of Faculty of Dentistry and Faculty of Pharmacy, Mahidol University on 25 April 2023 (COE.No.MU-DT/PY-IRB 2023/018.2504). SimOL was used as a mandatory task of an oral lesion course for fourth year dental undergraduates enrolled in the Doctor of Dental Surgery Program at the Faculty of Dentistry, Mahidol University. The learners were divided into two groups to ensure that adequate technical support was provided for all participants using SimOL simultaneously. Given the absence of prior confirmation regarding the effectiveness of the game in comparison to a lecture-based approach, a prerecorded lecture was retained alongside the game in the instructional design. Consequently, a waiver of informed consent was approved by the Institutional Review Board of Faculty of Dentistry and Faculty of Pharmacy, Mahidol University, as all students were required to complete the game as a part of their learning.

## Results

### Research participants

**Fig. 3:**
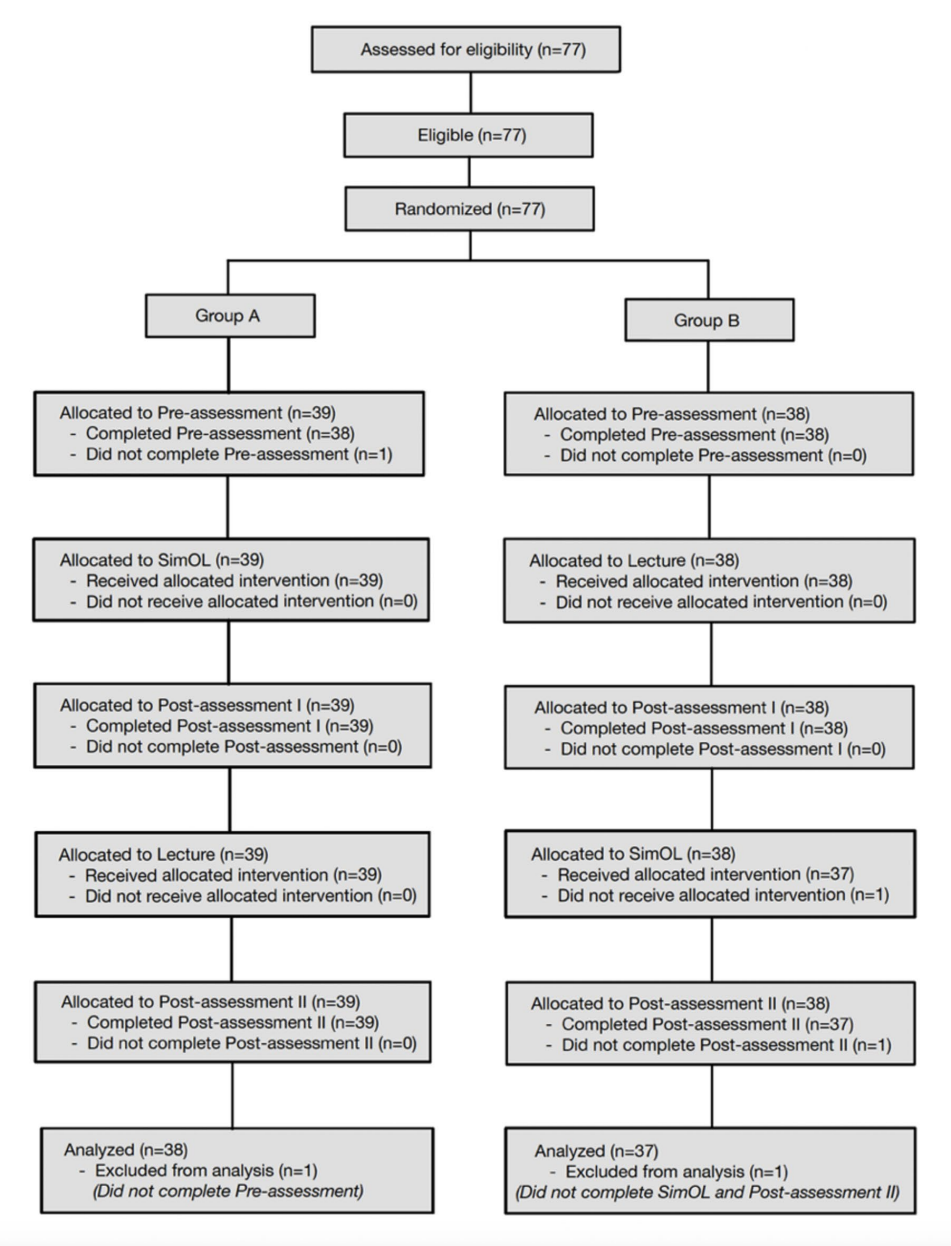
Diagram illustrating participant flow in this crossover randomized control trial.

A total of 77 dental undergraduates participated in the learning tasks, which were randomized into Group A (*n* = 39) and Group B (*n* = 38). However, two of them did not complete at least one of the assigned tasks within the arranged schedule. Consequently, only 75 students were included in this research (Group A: *n* = 38 and Group B: *n* = 37), as presented in Fig. 3.

### Knowledge improvement

There were statistically significant differences in scores among the three assessments for both groups [Group A: F(2, 74) = 53.126, *P* < 0.001; Group B: F(8, 72) = 45.643, *P* < 0.001], as reported by the repeated measure ANOVA analysis (Table 1).

**Table 1:** Repeated measure ANOVA of the scores from the three knowledge assessments.

	Source	Sum of Squares	DF	Mean square	F-value	*P*-value
**Group A**	**Assessment scores**	211.421	2	105.711	53.126	< 0.001
	**Error**	147.246	74	1.990		
**Group B**	**Assessment scores**	195.297	2	97.649	45.643	< 0.001
	**Error**	154.036	72	2.139		
Note: The full scores of all knowledge assessments were 15. The significance level was taken at *P* < 0.05. Abbreviation: DF, degrees of freedom.						

According to the Bonferroni post-hoc test, there was statistically significant improvement of the assessment scores among participants from both groups after completing the first instructional approaches, whether it was the game or the lecture (*P* < 0.001), as demonstrated in Table 2. There were significant increases in the assessment scores from 8.71 (SD = 2.05) to 11.47 (SD = 1.80) and from 8.57 (SD = 2.10) to 11.54 (SD = 1.59) in Group A and Group B, respectively. However, no statistically significant differences were found between the scores of Post-assessments I and II in both groups (*P* > 0.05).

**Table 2:** Bonferroni post hoc test for pairwise comparisons.

	Assessments	Mean (SD)	Comparison	Mean difference (SD)	*P*-value
**Group A**	**Pre-assessment**	8.71 (2.05)	Post-assessment I Post-assessment II	−2.76 (2.17) −3.00 (1.89)	< 0.001 < 0.001
	**Post-assessment I**	11.47 (1.80)	Pre-assessment Post-assessment II	2.76 (2.17) −0.24 (1.91)	< 0.001 1.000
	**Post-assessment II**	11.71 (1.63)	Pre-assessment Post-assessment I	3.00 (1.89) 0.24 (1.91)	< 0.001 1.000
**Group B**	**Pre-assessment**	8.57 (2.10)	Post-assessment I Post-assessment II	−2.97 (2.39) −2.62 (1.92)	< 0.001 < 0.001
	**Post-assessment I**	11.54 (1.59)	Pre-assessment Post-assessment II	2.97 (2.39) 0.35 (1.86)	< 0.001 1.000
	**Post-assessment II**	11.19 (1.76)	Pre-assessment Post-assessment I	2.62 (1.92) −0.35 (1.86)	< 0.001 1.000
Note: The full scores of all knowledge assessments were 15. The significance level was taken at *P* < 0.05. Abbreviation: SD, standard deviation.					

### Comparison of knowledge assessment scores between two learning designs

No statistically significant differences in the scores between Group A and Group B were found in all assessments (*P* > 0.05), as presented in Table 3.

**Table 3:** Comparison of knowledge assessment scores between the two learning designs.

Assessments	Group A Mean (SD)	Group B Mean (SD)	Mean difference (95% CI)	*P*-value
**Pre-assessment**	8.71 (2.05)	8.57 (2.10)	0.14 (−0.81-1.10)	0.767
**Post-assessment I**	11.47 (1.80)	11.54 (1.59)	−0.07 (−0.85-0.72)	0.865
**Post-assessment II**	11.71 (1.63)	11.19 (1.76)	0.52 (−0.26-1.30)	0.187
Note: The full scores of all knowledge assessments were 15. The significance level was taken at *P* < 0.05. Abbreviation: SD, standard deviation; 95% CI, 95% confidence interval.				

### Satisfaction of users with SimOL

All research participants completed and returned the satisfaction questionnaire. Participants were likely to have positive perceptions toward the use of SimOL, with no statistically significant differences between the two groups (*P* > 0.05). ‘Perceived usefulness’ was perceived most positively in both groups, with a score of 4.10 out of 5 (SD = 0.46) in Group A and 4.09 (SD = 0.44) in Group B. In contrast, ‘Perceived enjoyment’ received the lowest positive rating, with the score of 3.57 (SD = 0.70) in Group A and 3.60 (SD = 0.59) in Group B. These findings were presented in Table 4.

**Table 4:** Comparison of user satisfaction between the two learning designs.

Perceptions	Group A Mean (SD)	Group B Mean (SD)	Mean difference (95% CI)	*P*-value
**Perceived usefulness**	4.10 (0.46)	4.09 (0.44)	0.01 (−0.19-0.22)	0.897
**Perceived ease of use**	4.05 (0.71)	3.99 (0.63)	0.06 (−0.25-0.37)	0.709
**Perceived enjoyment**	3.57 (0.70)	3.60 (0.59)	−0.03 (−0.33-0.27)	0.861
**Overall**	3.91 (0.49)	3.89 (0.45)	−0.02 (−0.20-0.23)	0.890
Note: User satisfaction on a five-point Likert Scale, ranging from ‘Strongly disagree’ (1) to ‘Strongly agree’ (5). The significance level was taken at *P* < 0.05. Abbreviation: SD, standard deviation; 95% CI, 95% confidence interval.				

## Discussion

SimOL has been shown to be an effective instructional strategy in dental education. The results retrieved in this research demonstrated knowledge improvement among participants after the game completion, as evidenced by the pre- and post-assessments. Knowledge acquisition and skill enhancement have emerged as a required aspect in the evaluation of serious games in healthcare education^12,30,31^. The findings among this cohort of dental undergraduates remained consistent with the previous research, even though they were enrolled in different academic cohorts^10^. Through a game cycle, users could gain knowledge from the role of failure^32,33^. According to SimOL, when learners could not respond appropriately to a simulated patient, various formats of immediate feedback would allow them to learn from their mistake, leading to the improvement of knowledge and skills. This research contributed to further strengthen the existing evidence of simulation-based serious games in teaching oral lesions as a valuable tool for a diverse range of learners.

This study indicated comparable levels of knowledge enhancement between serious game and pre-recorded lecture formats, aligning with prior research indicating no significant differences in knowledge gains between serious games and traditional instructional approaches^34–37^. Several factors likely contribute to these comparable outcomes between the SimOL and pre-recorded lecture formats, with prior knowledge emerging as a key consideration. For students with strong foundational understanding, serious games, which require higher-order cognitive processing aligned with Bloom’s taxonomy levels of application and analysis^38–40^, can be particularly engaging and effective for reinforcing and applying knowledge in complex scenarios. However, for students with limited prior knowledge, these higher-order cognitive requirements could lead to cognitive overload, potentially causing stress and diminishing learning efficiency^41^. In such cases, the structured nature of pre-recorded lectures may better support foundational knowledge building, as they enable learners to process content linearly and at a lower cognitive load. This variation in prior knowledge may help explain the lack of significant difference in knowledge outcomes between the two formats. Nevertheless, this does not imply that serious games are ineffective. Traditional lecture-based approaches may limit learner engagement due to their passive nature^42,43^, whereas serious games immerse participants in dynamic and interactive learning environments that foster experiential learning and support knowledge construction through active participation and higher-order cognitive skills^12,44–46^. Each method may therefore effectively cater to different learner needs, potentially balancing their overall impact on knowledge acquisition.

In the context of dental education, SimOL has potential to create virtual clinical environments for learners to have the opportunity to perform diagnostic assessments, explore diverse scenarios, and develop their confidence in future dental practice. Simulation technologies empower serious games to mimic real-world dental situations, thereby facilitating students to enhance their competence in secure and controlled learning environments^12,47^. Therefore, another essential attribute of simulation-based serious games is to replicate clinical scenarios, where learners can enrich their knowledge and skills without exposing themselves and real patients to any potential harm.

Regarding the entertaining aspect of SimOL, perceived enjoyment was rated significantly lower than perceived usefulness and perceived ease of use, highlighting an area for improvement. Although several game elements were integrated into SimOL, such as cartoon characters with dynamic facial expressions, immediate feedback, and achievement-based rewards^10^, the game structure might limit user engagement. Currently, students proceed through the game in a step-by-step which could feel restrictive. Increasing the degree of freedom could address this concern by enabling students to explore alternative pathways based on their decisions^48^. Additionally, incorporating branching storylines could encourage critical thinking and give students a sense of autonomy and control over their learning path^49^. Introducing collaborative elements, where students interact with peers rather than solely with the game system, could also enhance social engagement, fostering teamwork and discussion, which in turn could lead to higher enjoyment levels^25^. Such enhancements would need to be carefully incorporated into SimOL to ensure the experience is more enjoyable for users.

The integration of SimOL into the dental curriculum was investigated in this research to assess its potential to either replace or supplement a lecture-based approach. The results indicated no significant differences in Post-assessment I scores between the lecture and SimOL groups, and the subsequent learning methods did not lead to a noticeable increase in knowledge among learners. Based on these results, SimOL could potentially replace a lecture-based approach, given its interactive and engaging features, even though learners from both groups acquired knowledge at a similar level. However, this does not imply that only one approach should be used. Given that learners may have distinct educational preferences^50^, the availability of two instructional strategies would allow them to select the approach that best suits their preferred learning styles.

Although this research is the first study conducting a crossover randomized controlled trial to compare the effectiveness of two distinct educational approaches in teaching diagnosis and treatment planning in oral lesions, through the implementation of a serious game, there were a few limitations in conducting this trial. The implementation of a double-blind technique was impractical for this research, although the researcher responsible for assessing and analyzing the data remained blinded to mitigate the risk of bias. Given that both learning interventions were administered to the entire fourth-year dental student cohort (with only two students excluded), encompassing both genders and varying academic performance levels, the findings of this research hold the potential for application in other groups of dental undergraduates embarking on clinical training in oral medicine. Furthermore, as SimOL allows learners to explore, experiment, and apply knowledge in practical contexts, potentially deepening their understanding of real-world applications, future research should assess the transferability of competencies gained from the game to actual clinical practice.

## Conclusions

SimOL has demonstrated educational outcomes comparable to those of traditional lectures in enhancing the cognitive skills of dental undergraduates in diagnosing and treatment planning for oral lesions. While future development of SimOL is required to increase engagement and offer users more freedom within the game to create a more engaging experience, it has the potential to serve as a suitable replacement for traditional or pre-recorded lectures in the oral lesion course, given its interactive nature. However, the availability of both options would allow learners to choose the learning method that aligns with their foundational knowledge and preferences, offering greater flexibility in their educational experience.

## Data Availability

The data that support the findings of this study are available from the corresponding author, up-on reasonable request. The data are not publicly available due to information that could compromise the privacy of research participants.
